# Tau aggregation and increased neuroinflammation in athletes after sports-related concussions and in traumatic brain injury patients – A PET/MR study

**DOI:** 10.1016/j.nicl.2021.102665

**Published:** 2021-04-07

**Authors:** Niklas Marklund, Fredrik Vedung, Mark Lubberink, Yelverton Tegner, Jakob Johansson, Kaj Blennow, Henrik Zetterberg, Markus Fahlström, Sven Haller, Staffan Stenson, Elna-Marie Larsson, Anders Wall, Gunnar Antoni

**Affiliations:** a Department of Neuroscience, Neurosurgery, Uppsala University, Uppsala, Sweden; b Lund University, Skåne University Hospital, Department of Clinical Sciences Lund, Neurosurgery, Lund, Sweden; c Medical Physics, Uppsala University Hospital, Uppsala, Sweden; d Department of Surgical Sciences, Nuclear Medicine and PET, Uppsala University, Sweden; e Department of Health Sciences, Luleå University of Technology, Sweden; f Department of Surgical Sciences, Anesthesiology, Uppsala University, Sweden; g Institute of Neuroscience and Physiology, Department of Psychiatry and Neurochemistry, Sahlgrenska Academy at the University of Gothenburg, Mölndal, Sweden; h Clinical Neurochemistry Laboratory, Sahlgrenska University Hospital, Mölndal, Sweden; i UK Dementia Research Institute at UCL, London, United Kingdom; j Department of Neurodegenerative Disease, UCL Institute of Neurology, London, United Kingdom; k Department of Surgical Sciences, Radiology, Uppsala University, Uppsala, Sweden; l CIMC - Centre d’Imagerie Médicale de Cornavin, Place de Cornavin 18, 1201 Genève, Switzerland; m Department of Neuroscience, Rehabilitation Medicine PET Centre, Uppsala University Hospital, Uppsala, Sweden; n Department of Medicinal Chemistry, Uppsala University, Uppsala, Sweden

**Keywords:** Traumatic brain injury, Sports-related concussion, Inflammation, Tau, Positron emission tomography (PET)

## Abstract

Traumatic brain injury (TBI) and repeated sports-related concussions (rSRCs) are associated with an increased risk for neurodegeneration. Autopsy findings of selected cohorts of long-term TBI survivors and rSRC athletes reveal increased tau aggregation and a persistent neuroinflammation. To assess *in vivo* tau aggregation and neuroinflammation in young adult TBI and rSRC cohorts, we evaluated 9 healthy controls (mean age 26 ± 5 years; 4 males, 5 females), 12 symptomatic athletes (26 ± 7 years; 6 males, 6 females) attaining ≥3 previous SRCs, and 6 moderate-to severe TBI patients (27 ± 7 years; 4 males, 2 females) in a combined positron emission tomography (PET)/magnetic resonance (MR) scanner ≥6 months post-injury. Dual PET tracers, [^18^F]THK5317 for tau aggregation and [^11^C]PK11195 for neuroinflammation/microglial activation, were investigated on the same day. The Repeated Battery Assessment of Neurological Status (RBANS) scores, used for cognitive evaluation, were lower in both the rSRC and TBI groups (p < 0.05). Neurofilament-light (NF-L) levels were increased in plasma and cerebrospinal fluid (CSF; p < 0.05), and serum tau levels lower, in TBI although not in rSRC. In rSRC athletes, PET imaging showed increased neuroinflammation in the hippocampus and tau aggregation in the corpus callosum. In TBI patients, tau aggregation was observed in thalami, temporal white matter and midbrain; widespread neuroinflammation was found e.g. in temporal white matter, hippocampus and corpus callosum. In mixed-sex cohorts of young adult athletes with persistent post-concussion symptoms and in TBI patients, increased tau aggregation and neuroinflammation are observed at ≥6 months post-injury using PET. Studies with extended clinical follow-up, biomarker examinations and renewed PET imaging are needed to evaluate whether these findings progress to a neurodegenerative disorder or if spontaneous resolution is possible.

## Highlights

• Traumatic brain injury (TBI) leads to axonal injury and an inflammatory response.
• Repeated sports-related concussions (rSRC) are linked to neurodegeneration.
• We studied tau aggregation and neuroinflammation in rSRC and TBI using PET/MRI.
• In young rSRC and TBI patients, tau aggregation and neuroinflammation was increased.
• PET useful when studying the long-term consequences of rSRC and TBI.

## Graphical abstract

**Figure:**
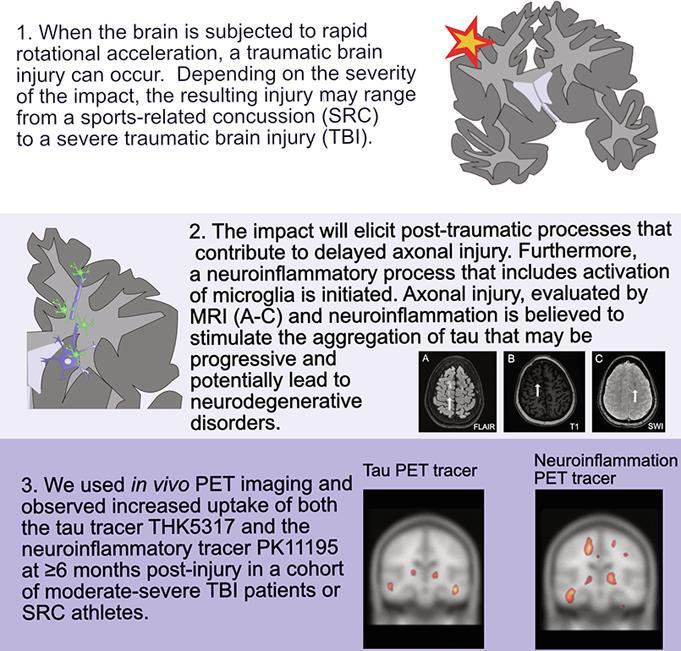

## 1 Introduction

A sports-related concussion (SRC) is caused by an external force transmitted to the head, and results in transient neurological symptoms (McCrory et al., 2017). Whilst approximately 85–90% of concussed athletes recover within the first post-injury weeks, a subset develops prolonged symptoms. In addition, repeated SRCs (rSRC) are associated with neurodegeneration and may lead to an earlier onset of Alzheimeŕs disease (AD) (Manley et al., 2017).

Increasingly, athletes with a history of repeated SRCs are at autopsy found to display chronic traumatic encephalopathy (CTE), a neurodegenerative tauopathy characterized by aggregation of phosphorylated tau protein (McKee et al., 2016). The vast majority of CTE cases have been observed in elderly athletes, with only a few observations of tau aggregation in teenagers at autopsy (Tagge et al., 2018). Furthermore, there is a striking male predominance with very few females found to display the clinical and histopathological features of CTE. The pathogenesis of CTE remains unknown, although neuroinflammation and axonal pathology may be contributing factors (Johnson et al., 2013, Johnson et al., 2012, Wilson et al., 2017, McKee et al., 2013).

Severe traumatic brain injury (TBI) is a chronic disease process leading to progressive white matter atrophy, linked to a persistent neuroinflammation (Johnson et al., 2013), and an increased risk for neurodegenerative disorders (Wilson et al., 2017). Tau aggregation in the form of neurofibrillary tangles is observed at autopsy following TBI, although only in those with several months survival times post-injury (Johnson et al., 2012). While an SRC is defined as a mild TBI, there may be important pathophysiological differences between SRC and single, severe TBI.

In TBI and SRC, the shearing forces induced at time of head impact could result in axonal and white matter pathology that may be progressive. The phosphorylated tau (p-tau) pathology observed in CTE has been linked to axonal distortion and degeneration (McKee et al., 2013), and p-tau is increased in injured axons with a similar distribution and temporal profile as other markers of axonal injury (Caprelli et al., 2018). In CTE cases, axonal disruption was observed underlying cortical sulci showing tau pathology (Holleran et al., 2017). Neuroinflammation is known to contribute to the development of neurodegeneration in e.g. TBI and CTE (Johnson et al., 2013, Erturk et al., 2016), and in AD, microglial activation occurs before tau aggregation, neuronal loss, and behavioral dysfunction (Dani et al., 2018). Human and experimental TBI studies have also shown persistent microglial activation and tau pathology (Loane et al., 2014, Ramlackhansingh et al., 2011), which coincide with axonal degeneration (Johnson et al., 2013). Thus, there is much evidence suggesting a link between axonal injury, neuroinflammation and tau aggregation.

However, since most evidence for this hypothesis is obtained from autopsy and experimental studies, methods to monitor neuroinflammation and tau aggregation *in vivo* are needed. Recently, tau aggregation was found in elderly athletes or in severe TBI patients using novel positron emission tomography (PET) tau-tracers (Stern et al., 2019, Barrio et al., 2015, Dickstein et al., 2016, Lee et al., 2018, Small et al., 2013, Takahata et al., 2019, Gorgoraptis et al., 2019). To study tau aggregation and neuroinflammation in young cohorts of moderate-severe TBI patients and symptomatic rSRC athletes, we used dual PET tracers in combination with biomarkers, neuropsychological evaluation and 3T MR scanning.

## 2 Methods

### 2.1 Study participants

Written informed consent was obtained from each participant. The Regional Research Ethics Committee in Uppsala granted permission for the study (Dnr 2015/012), conducted in accordance with the ethical standards given in the Helsinki Declaration.

Twelve athletes (6 males and 6 females; mean age 26 ± 7 years) with repeated sports-related concussions (rSRC) and ≥6 months duration of post-concussion symptoms, diagnosed as having post-concussion syndrome (PCS) according to the 4th edition of the Diagnostic and Statistical Manual of Mental Disorders (American Psychiatric Association, 2000) were recruited from across Sweden through word of mouth, social media posts, or e-mail notifications. The recent definition of concussion was used (McCrory et al., 2017), and all athletes had previous evaluation by CT and/or MR scanning (Table 1, Table 2).

**Table 1:** Characteristics of study participants- controls, TBI patients and rSRC athletes.

Group	Controls	rSRC	TBI
Patients (n)	9	12	6
Age (years)	26 ± 5 (20–34)	26 ± 7 (20–43)	27 ± 7 (21–40)

*Gender (n)*			
Male	4	6	4
Female	5	6	2
Number of SRC	0	6 (3–10)	–
Time since last SRC (months)	–	23 (6–132)	–

*Symptoms (SCAT)*			
SSS		48.5 (3–91)	
NOS		18 (2–22)	
Time since TBI (months)	–	–	19 ± 8 (10–29)
Length of hospital stay (days)	–	–	17 ± 9 (8–29)

*Injury type on CT/MR*			
Contusion	–	–	4
DAI	–	–	2

*Injury mechanics(n)*			
Fall	–	–	3
Motor vehicle accident	–	–	3
Sports activity (n)	–	12	0
Ice Hockey	–	8	–
Soccer	–	2	–
Downhill skiing	–	2	–

*Neurologic status*			
GCS at admission	–	–	12 (5–14)
GCS at discharge	–	–	14 (8–15)

Characteristics of study participants. In athletes, concussion symptoms were assessed by the Sport Concussion Assessment Tool (SCAT) 3. The symptom evaluation score lists 22 symptoms with a severity range of 0 - 6 where the symptom severity score (SSS) is the sum of all symptom scorings (range 0-132). The Number of Symptoms (NOS) is the sum of each symptom with a severity score between 1 and 6 (range 0-22). rSRC= Sports-related concussion; TBI=Traumatic Brain Injury; SCAT= sports concussion assessment tool; SSS= symptom severity score; NOS= number of symptoms; DAI = Diffuse Axonal Injury; CT= Computed Tomography; MR= Magnetic Resonance; GCS= Glasgow Coma Scale; Nonparametric data (number of SRCs, time since last SRC, SSS, NOS, GCS) is presented as 21 medians and range, parametric data (age, time since TBI and length of hospital stay) as means ± standard deviations (SD).

**Table 2:** Detailed characteristics of included athletes.

Age (years)	Sport	# of SRC	Last SRC (month)	NOS	SSS	Sex	RBANS	CSF NF-L (pg/mL)	CSF Tau (pg/mL)
24	Soccer	7	6	9	21	F	97	280	194
20	Alpine skiing	4	22	18	46	F	92	268	60
32	Soccer	4	6	20	51	F	116	388	223
28	Alpine skiing	10	6	18	70	F	57	264	122
24	Ice hockey	5	15	20	60	F	90	350	153
19	Ice hockey	4	28	12	34	F	66	284	182
43	Ice hockey	10	60	5	9	M	78	–	–
41	Ice hockey	10	120	22	91	M	80	410	176
21	Ice hockey	3	54	19	53	M	69	461	173
23	Ice hockey	6	24	17	35	M	63	448	478
27	Ice hockey	6	36	2	3	M	87	470	165
26	Ice hockey	7	18	22	71	M	65	260	213

Characteristics of the athletes with post-concussion syndrome participating in the study, including individual cerebrospinal fluid (CSF) neurofilament-light (NF-L) and tau levels (in pg/mL) . # = number; SRC = Sport Related Concussion; NOS = Number of Symptoms; SSS = Symptom Severity Score; F = Female; M = Male.

Six patients (4 males and 2 females; mean age 27 ± 7 years) with a moderate (defined as Glasgow Coma Scale (GCS) score 9–13, loss of consciousness ≥5 min and/or focal neurologic deficits (Ingebrigtsen et al., 2000) – to severe (GCS score ≤8) TBI and treated at the neurocritical care unit ≥6 months previously at the department of Neurosurgery, Uppsala University hospital (Table 1, Table 3) were conveniently recruited.

**Table 3:** Detailed characteristics of included TBI patients.

Gender	Age	Pathology	GCS – admission	GCS – discharge	Time since injury (month)	ICP – monitoring	NICU (days)	GOS	RBANS index
M	31	Bifrontal Contusions	13	14	10	No	1	5	82
M	40	tSAH, contusion	8	14	22	yes	14	5	86
M	23	DAI, tSAH	11*	15	12	No	29	4	50
M	25	Contusion	13	15	25	No	1	5	102
F	21	Contusion, SDH	14	15	14	No	3	5	89
F	23	DAI, tSAH	5	8	29	yes	18	4	40

Patient characteristics for the patients with traumatic brain injury (TBI) included in the study. GCS = Glasgow Coma Scale; ICP = Intracranial pressure; NICU = Neuro-intensive care unit; RBANS = Repeatable Battery for the Assessment of Neuropsychological Status; M = Male; F = Female; SDH= subdural hematoma; tSAH = traumatic Subarachnoid Haemorrhage; DAI = Diffuse Axonal Injury; GOS= Glasgow Outcome Scale; * this patient deteriorated to CGS 6 during neurocritical care.

Nine healthy, age-matched, non-contact sports-active and non-smoking controls (4 males, 5 females; mean age 26 ± 5 years) without history of TBI, SRC or neurological disorders were recruited through word of mouth or via Clinical Trial Consultants, Uppsala, Sweden.

### 2.2 Cognition, outcome and symptoms

On the day of and prior to the PET/MR investigation, a licensed neuropsychologist performed the Repeated Battery Assessment of Neurological Status (RBANS), a neurocognitive battery evaluating attention, language, memory, and visuospatial/constructional skills (McKay et al., 2007). The RBANS protocol was used to assess cognitive outcome in all participants. The Glasgow outcome scale (GOS) was used to assess outcome in TBI patients. In athletes, concussion symptoms were assessed by the Sport Concussion Assessment Tool (SCAT)-3, see Table 1. These evaluations were all performed the day of the PET/MR studies.

### 2.3 Biomarker sampling

At the time of PET/MR imaging, serum and plasma samples were collected by venipuncture. Plasma tau concentration was measured using the Human Total Tau 2.0 kit and the Single molecule array (Simoa) HD-1 analyzer according to instructions from the manufacturer (Quanterix, Lexington, MA, USA). Serum NF-L concentration was measured using an in house Simoa assay. CSF was collected by routine lumbar puncture after the PET/MR imaging. CSF NF-L concentration was measured using a commercially available NF-Light kit (UmanDiagnostics, Umeå, Sweden) and CSF tau concentration by INNOTEST ELISA (Fujirebio, Ghent, Belgium). In the control group, ethical permission for cerebrospinal fluid (CSF) sampling was not granted in Uppsala. Instead, CSF control samples from 19 neurologically healthy controls (mean age 26, range 21–35 years old; 14 male, 5 females) without known history of TBI or SRC available at the Department of Psychiatry and Neurochemistry, University of Gothenburg were used (Shahim et al., 2016).

### 2.4 PET/MR

Each study participant was instructed to refrain from coffee, tea, energy drinks and nicotine from mid-night the day, and alcohol the whole day, prior to the PET/MR scan.

All subjects underwent PET scans after bolus injection of the neuroinflammation tracer [^11^C]PK11195 (3.2 ± 0.4 MBq/kg) and later the tau tracer [^18^F]THK5317 (4.7 ± 0.3 MBq/kg) on a Signa PET-MR scanner (GE Healthcare, Waukesha). PET images were reconstructed into 22 frames (6 × 10 s, 3 × 20 s, 2 × 30 s, 2 × 60 s, 2 × 150 s, 4 × 300 s, 3 × 600 s) using ordered-subset expectation maximization (4 iterations, 28 subsets) applying all appropriate corrections and a 5 mm Hanning postfilter. Attenuation correction was based on a zero-echo time MR-sequence (Sousa et al., 2018).

MRI was performed during PET-scanning with multiple sequences including 3D T1-weighted gradient echo (GRE), 3D T2-weighted fluid attenuated inversion recovery (FLAIR), 3D T2-weighted fast spin echo, diffusion weighted and susceptibility weighted scans. Volumetric measurements of hippocampus bilaterally and corpus callosum were acquired using the Freesurfer processing pipeline (version 6.0, http://surfer.nmr.mgh.harvard.edu) using T1w- and T2-FLAIR images. Corpus callosum was segmented into five segments and total volume was obtained by adding the volume of each segment. Volumes are given in mm^3^.

### 2.5 Image analysis – PET

All dynamic PET images were corrected for within scan movement utilizing Voiager 4.0.7 (GE Healthcare) software. The T1-weighted MR volumes were co-registered to the sum of the first 5-min PET images using Statistical Parametric Mapping 8 (SPM8; Wellcome Trust Centre for Neuroimaging Institute of Neurology, University College of London, UK) and segmented into grey and white matter.

Parametric images of [^18^F]THK5317 binding potential (BP_ND_), a measure of tau aggregation, were computed using the reference Logan method (Logan et al., 1996) using cerebellar grey matter cerebellum as reference tissue. A grey-matter cerebellum VOI was defined on T1-MR images using an automatic probabilistic atlas (PVElab (Svarer et al., 2005) and projected over all frames of the dynamic PET scans to obtain the reference tissue time-activity curve. In addition, data were analysed using a basis function implementation of the simplified reference tissue model (Gunn et al., 1997) to obtain relative cerebral blood flow (rCBF) images depicting CBF relative to CBF in grey matter cerebellum.

For [^11^C]PK11195, BP_ND_ images, showing translocator protein expression as a measure of neuroinflammation, were computed after dynamic denoising of the PET images using RPM including blood volume parameter (Yaqub et al., 2012). Since no anatomically defined reference tissue lacking 18-kDA translocator protein (TSPO) exists, a supervised clustering method was used to obtain the reference tissue time-activity curve (Turkheimer et al., 2007).

All subjects’ T1-MR-volumes were subsequently spatially normalized to the MNI-template with SPM12, considered to provide better spatial normalization than SPM8, and the same transformations applied to the parametric PET images. After applying a brain mask and additional smoothing with an 8 mm gauss filter, differences between groups were assessed using t-tests at the voxel level with two-sample t-tests. A T-value of 4 was used as significance threshold for the images. For a subsequent analysis at the VOI level based on significant clusters, a T-threshold of 2 and a cluster size threshold of 50 was applied. Total tau load (BP_ND_ × cm^3^) in cortical grey matter, white matter, and subcortical grey matter (putamen, thalamus and caudate nucleus) was computed by summation of all voxels [^18^F]THK5317 BP_ND_ values multiplied by voxel volume. In addition, the number of voxels with BP_ND_ > 0.5 in grey and white matter, as well as the skewness of the distribution of BP_ND_ values over all voxels in grey and white matter were computed.

### 2.6 Image analysis – MRI

Structural MRI with T1-, T2- and susceptibility weighted images were evaluated by two experienced neuroradiologists (Suppl. Fig. 2)

### 2.7 Statistical analysis

Statistical analysis was performed with GraphPad Prism version 8.1.0 for Windows, GraphPad Software, San Diego, California USA. Normality of data was evaluated by Shapiro-Wilk. Non-parametric data is presented as medians, interquartal range (IQR) and range, while parameteric data as means ± standard deviations (SD). The non-parametric Kruskal-Wallis was used to investigate differences between more than two unpaired groups, followed by the Mann-Whitney *U* test, e.g. RBANS, plasma tau, CSF tau, plasma NF-L and CSF NF-L. Derived P-values are two-sided and presented as exact values, P-values ≤ 0.05 were considered significant.

## 3 Results

### 3.1 Study participants

#### 3.1.1 Athletes with repeated sports-related concussion (SRC) and post-concussion syndrome (PCS)

The athletes were subjected to PET/MR imaging at a median of 23 (range: 6–132) months following their last SRC. They had attained a median of 6 sports-related concussion (range 3–10) and had high symptom severity on SCAT evaluation (Table 1). Three athletes were on anti-depressant medication (one on sertraline, two on amitriptyline) while not on any other treatment for psychiatric disorders.

#### 3.1.2 TBI patients

Of the TBI patients, four had a good clinical recovery (GOS 5) and two a moderate disability (GOS 4). They underwent PET/MR Imaging 19 ± 8 (range 10–29) months post-injury. No TBI patient had any known psychiatric of psychological disorder prior to the injury. Their characteristics are shown in Table 1, Table 3.

#### 3.1.3 Controls

**Supplementary Fig. 1:**
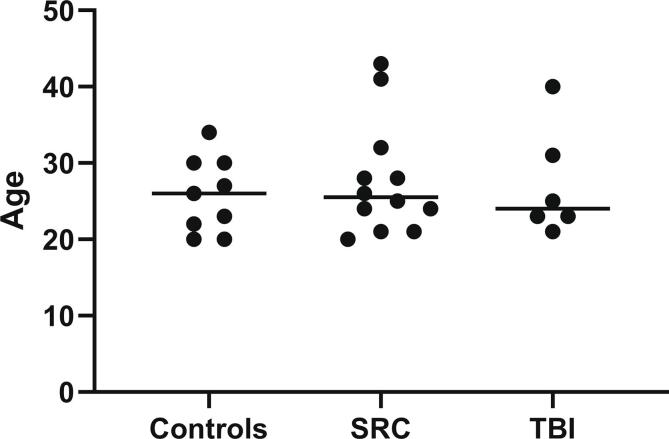
Age distribution of included study participants. Age distribution in the repeated sports-related concussion (rSRC), traumatic brain injury (TBI) and control groups is shown.

**Supplementary Fig. 2:**
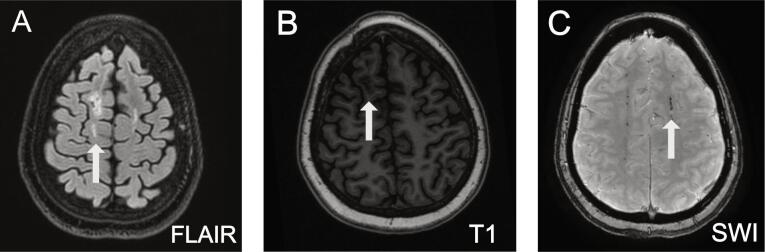
Examples of MR sequences used in the study. Examples of 3T magnetic resonance (MR) images obtained using the PET/MR Imaging technique in a patient who sustained a TBI ≥ 6 months previously. A is showing a FLAIR (Fluid-attenuated inversion recovery) image, B a T1 3Dgre and in C, SWI, Susceptibility-Weighted Image. White matter changes indicating diffuse axonal injury are observed (arrows). All athletes with repeated sports-related concussions and post-concussive symptoms had normal 3TMR scans using routine MR sequences (not shown).

Nine controls (mean age 26.5 ± 5; range 20–34), four males and five females, were included (Table 1). Two were excluded; one previous Thai boxing participant was found to have a hippocampal traumatic lesion. The second was an outlier >2.5 SD in all PET-data and also in Arterial Spin Labeling blood flow measurements (data not shown). Age distribution for all participants shown in Suppl. Fig. 1.

### 3.2 Outcome

**Fig. 1:**
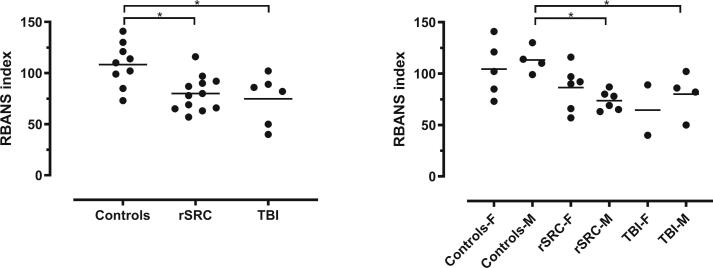
Cognitive function in TBI and rSRC.

The TBI patient and rSRC athlete groups performed worse on the RBANS, a cognitive screening test (controls: 105.5 ± 21; rSRC: 80 ± 17; TBI: 75 ± 24; controls to rSRC: p = 0.006; controls to TBI: p = 0.03; Fig. 1). Using a criterion of 2 SD from the mean, 2/6 TBI patients and 5/12 rSRC athletes were clearly impaired while others performed at a level of healthy controls (Fig. 1). Male rSRC athletes and male TBI patients had lower RBANS scores than male controls (Fig. 1). GOS and SCAT results are presented in Table 1.

### 3.3 Blood and CSF biomarkers

**Fig. 2:**
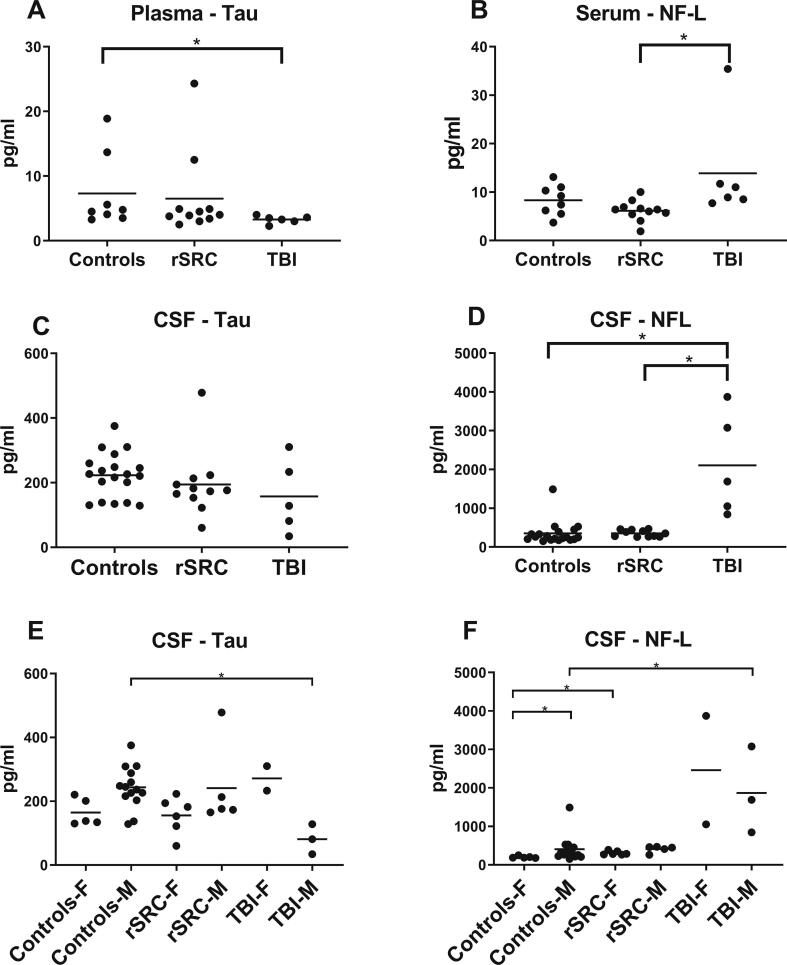
Tau and neurofilament-light in CSF and blood. Biomarkers for axonal injury/neurodegenerastion (Neurofilament-light; NF-L and Tau) in blood (A-B) and cerebrospinal fluid (CSF; C-D). CSF samples from healthy controls were obtained from a separate cohort (N=19, 14 males, 5 females; mean age 26, range 21-25 years old), as previously published. (A, C) Symptomatic athletes who had attained repeated sports-related concussion (rSRC) had similar plasma tau levels when compared to controls. In contrast, plasma tau levels were significantly lower in traumatic brain injury (TBI) patients when compared to both healthy controls and rSRC athletes. In the CSF, the median tau levels tended to be lower in both concussed athletes and TBI patients than those of healthy controls. (B,D) Symptomatic rSRC athletes had similar levels of serum and CSF NF-L when compared to controls. In contrast, TBI patients had increased CSF and serum NF-L when compared to controls (*p<0.05). (E,F) CSF biomarker levels in male (M) and female (F) participants, significant changes indicated by *.

Plasma tau levels, measured in samples collected up to 132 months post-injury, were lower in TBI patients when compared to healthy controls (Controls: 4.7 (3.3–18.9) pg/mL; rSRC: 4.0 (2.5–24.3) pg/mL; TBI: 3.4 (2.3–4.0) pg/mL; p = 0.02). Serum neurofilament-light (NF-L) plasma levels were higher in the TBI group when compared to the rSRC athletes (controls: 8 (4–13) pg/mL; rSRC: 6 (2–10); TBI: 10 (8–35); rSRC to TBI p = 0.002; Fig. 2).

One TBI patient and one rSRC athlete declined CSF sampling. CSF t-tau levels tended to be lower in the TBI group when compared to controls (Controls: 226 (129–375) pg/mL; rSRC: 176 (60–478) pg/mL; TBI: 128 (34–310) pg/mL; p = 0.16). NF-L CSF levels were increased in the TBI group when compared to healthy controls and rSRC athletes (controls: 258 (153–1491) pg/mL; rSRC: 350 (260–470) pg/mL; TBI: 1691 (844–3873) pg/mL; control to TBI p = 0.0002; rSRC to TBI p = 0.001; Fig. 2). Moreover, CSF tau levels were lower in male TBI patients, and CSF NF-L levels higher, when compared to male controls (Fig. 2 E-F). CSF NF-L levels were also higher in female rSRC athletes when compared to female controls (Fig. 2F).

### 3.4 MR imaging

**Fig. 3:**
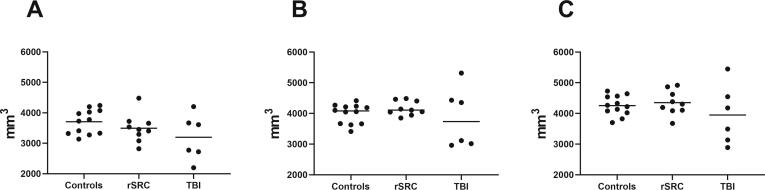
Volumes of the corpus callosum (A), and of the left (B) and right (C) hippocampus.

In controls, a non-specific small white matter signal was found in one subject. In the rSRC subjects, one small non-specific white matter lesion could be seen in two subjects and another had a minor pituitary lesion. In the TBI-group, findings of contusion rests (n = 4) and lesion suggestive of diffuse axonal injury (DAI; n = 2) were observed (Data not shown). Examples of the sequences used are shown in Suppl. Fig. 1. Using volumetric assessment, areas of increased tracer uptake (hippocampus, corpus callosum) were analysed. No significant differences among the groups were observed (Fig. 3).

### 3.5 PET imaging

**Fig. 4:**
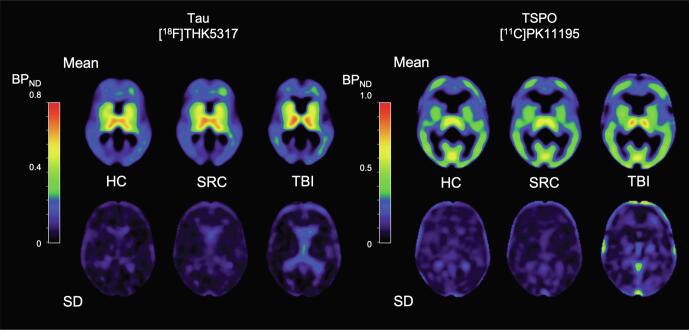
Parametric images of [^18^F]THK5317 and [^11^C]PK11195 BP_ND_.

**Fig. 5:**
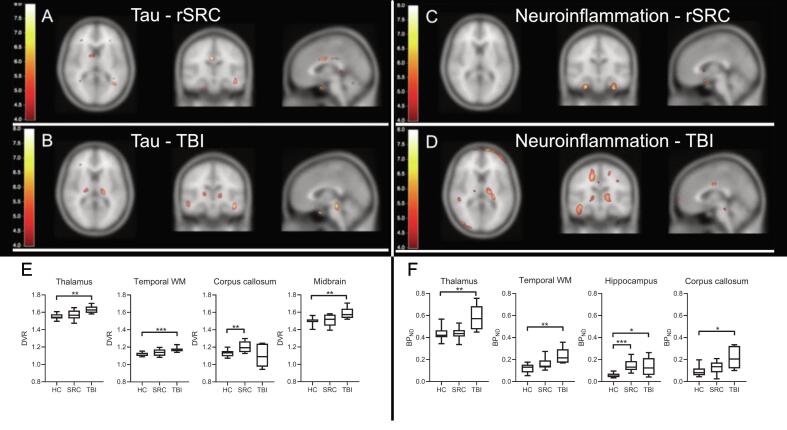
Increased neuroinflammation and tau aggregation in TBI and rSRC.In upper panels (A-B), group-wise comparisons of tau aggregation using [18F]THK5317 PET, and neuroinflammation/microglial activation using [11C]PK11195 in young adult repeated sports-related concussion (rSRC) athletes scanned ≥6 months following their last concussion, and young adult TBI patients scanned ≥6 months post-injury. (A, C) In symptomatic rSRC athletes, increased binding of the tau tracer was increased in the corpus callosum. The uptake was also increased subcortically, including the medial temporal lobe unilaterally. The binding of the [11C]PK11195 neuroinflammation tracer was increased in the medial temporal lobes. (B, D) Clusters of significantly elevated tau and neuroinflammation/microglial activation was observed in the thalamus, temporal lobe white matter, internal capsule and midbrain (tau only) of TBI patients. Color bars represent T values from 4 to 8.(E-F) Box plot representation in selected brain regions, based on >50 voxel clusters with each having a T value ≥ 2, i.e. ≥ 2 standard deviations. (E) Distribution volume ratios (DVR) for THK5317 shows increased tau aggregation in the thalamus, temporal white matter (WM) and midbrain of TBI patients, and in the corpus callosum of symptomatic rSRC athletes when compared to controls. (F) Increased binding potential (BPnd, TSPO) for PK11195 is observed in the thalamus, temporal WM and corpus callosum of TBI patients, and in the hippocampus of symptomatic rSRC athletes as well as TBI patients, when compared to controls. *=p<0.05, **=p<0.01, ***=p<0.001. TBI; Traumatic brain injury; rSRC; repeated Sports-related concussion. HC; healthy controls.

Fig. 4 shows mean [^18^F]THK5317 and [^11^C]PK11195 BP_ND_ images for controls, rSRC and TBI patients, depicting tau and TSPO expression, as well as corresponding standard deviation images. In symptomatic rSRC athletes, a voxel-wise *t*-test showed clusters of significantly increased [^18^F]THK5317 binding in the corpus callosum and subcortically including the medial temporal region, and [^11^C]PK11195 binding in the medial temporal lobes. In TBI, elevated tau and TSPO binding was observed in the thalamus, temporal lobe white matter and midbrain (tau only; Fig. 5). Significant group differences in total tau load were found between healthy controls and rSRC in subcortical grey matter (SRC 7.5 ± 0.9, controls 6.7 ± 0.5, p = 0.038), although not between TBI patients and controls. No significant differences in number of voxels with [^18^F]THK5317 BP_ND_ > 0.5 or skewness in BP_ND_ distribution were found.

## 4 Discussion

We used dual PET tracers for tau and neuroinflammation/microglial activation and found increased tau aggregation and neuroinflammation/microglial activation in young, symptomatic athletes with a history of ≥3 sports-related concussions (rSRC) and in a cohort of young adult patients with a single moderate-severe TBI. This is the first tau PET study evaluating TBI patients and rSRC athletes in such young cohorts (mean age 25–27 years old) (Small et al., 2013, Mohamed et al., 2019, Stern et al., 2019, Barrio et al., 2015, Dickstein et al., 2016). The rSRC athletes met the criteria for post-concussion syndrome(American Psychiatric Association, 2000) and had symptom scores higher than previously investigated normality scores (Hänninen et al., 2016). On a group level, both the athletes and TBI patients had increased tau aggregation and neuroinflammation/microglial activation. The imaging was performed 6 months up to several years following the last SRC or TBI implying persisting pathology at prolonged post-injury time points, supported by the increased CSF and serum NF-L levels. Studies using extended clinical follow-up and biomarker examinations are needed to address whether these changes progress over time, as suggested in clinical and experimental studies (Zanier et al., 2018), or whether spontaneous resolution is possible. TBI patients, but not rSRC athletes, showed increased CSF and serum NF-L levels and reduced plasma tau levels implying ongoing injury processes detected by biomarker sampling. The significance of the reduced CSF and serum tau levels observed in the TBI groups may be related to decreased activity-dependent release of tau from neurons (Yamada et al., 2014). In addition, some non-AD disease tauopathies show tendencies towards lower CSF tau levels (Hall et al., 2012). However, the cause-and-effect relationships need to be examined in future studies. Of note, the TBI patients and rSRC athletes represent two distinct groups. Although both athletes and TBI patients had cognitive impairment on neuropsychological testing, only TBI patients had findings of traumatic lesions on routine 3TMR Imaging.

We used a combined PET/MR scanner in a single imaging session. The atlas analysis and calculation provided a measure of the density of available targets, reflecting the binding sites for [^18^F]THK5317 or a measure of binding sites for [^11^C]PK11195. Individual cases may differ from the reference group, and larger case series are needed to establish pathological individual values (Stern et al., 2019). The [^18^F]THK5317 tracer has a beta-sheathed configuration and can bind both to phosphorylated and non-phosphorylated tau (Mathis et al., 2017). Thus, it may have some unspecific white matter binding. However, the within-group variability in the control, TBI and SRC groups was surprisingly low. The structurally related tracer [^18^F]THK5351 may bind to monoamine oxidase-B (MAO-B), present in astrocytes (Ng et al., 2017) and thus the specificity of this tracer has been questioned. A recent in vitro study of the racemic compound [^3^H]THK5117 showed relatively low affinity to MAO-B indicating that competitive binding of the (S)-enantiomer [^18^F]THK5317 would not present a problem at concentrations encountered during clinical PET studies (Lemoine et al., 2017) Very recently, the novel [^11^C]PBB3 tracer, suggested not to cross-react with MAO-A or MAO-B, was evaluated in TBI patients (Takahata et al., 2019).

We observed an increased [^18^F]THK5317 binding in white matter regions, particularly in the corpus callosum of rSRC athletes and in the thalamus, temporal white matter and midbrain of TBI patients. The evaluated cohorts had no clinical features associated with CTE, and the distribution of the tau PET tracer is also different from that observed in CTE. Irregular p-tau aggregates around small vessels at the depths of the cortical sulci is considered pathognomonic in CTE, but supporting abnormal p-tau immunoreaction in various brain regions such as subcortical nuclei, the hippocampus and the periventricular region may also be found (McKee et al., 2016). A hierarchical progression of pathology has also been suggested (McKee et al., 2014) and in late survivors of TBI, paired helical fragments were found across the medial temporal lobe (Zanier et al., 2018).

As noted previously, persistent white matter pathology in combination with the increased microglial activation suggested here may results in exacerbation of tau pathology, and repeat PET imaging will be needed to establish progression over time. We also observed increased tau uptake in the corpus callosum, a white matter region susceptible to injury following SRC and TBI (Chamard et al., 2016, Wu et al., 2020). Under normal condition, tau is soluble and abundant in axons. When hyperphosphorylated, *e.g*. from trauma, it dissociates from microtubules and may aggregate into paired helical filaments (PHFs) and neurofibrillary tangles (Katsumoto et al., 2019). [^18^F]THK5117, the racemate of the more active S-enantiomer THK5317, binds to paired helical filaments and neuropil threads found in white matter (Okamura et al., 2005, Harrington et al., 1994). Thus, it is plausible that [^18^F]THK5117 accumulation in the corpus callosum reflects tau aggregation.

As noted, previous tau PET studies evaluated retired and markedly older athletes (Takahata et al., 2019, Gorgoraptis et al., 2019, Stern et al., 2019, Barrio et al., 2015, Dickstein et al., 2016). Recently, symptomatic male former American football players 40–69 years old had increased tau levels on PET imaging in brain regions affected by CTE (Stern et al., 2019). Using Flortaucipir, increased tracer binding was observed in a small and heterogeneous TBI cohort (age range 29–72 years) (Ramlackhansingh et al., 2011), and using the [^11^C]PBB3 tracer in TBI the median age was 46 years old (Takahata et al., 2019).

In addition, when a 39-years old athlete with 22 reported concussions and clinical features consistent with CTE was evaluated, tau tracer uptake in subcortical white matter regions was observed. Several years later, autopsy confirmed CTE in this individual (Dickstein et al., 2016). In these studies, increased tau aggregation was consistently observed in the medial temporal lobes and the amygdala, but also cortically in the frontal and/or parietal lobes, consistent with the distribution of p-tau in CTE. Thus, future studies are needed to evaluate whether the tau aggregation observed in our present study progress into a CTE-like distribution, as suggested in the experimental setting (Zanier et al., 2018).

Only rarely have young athletes attaining repeated SRCs been evaluated at autopsy. In a cohort of 4 young athletes who died shortly after SRC, focal p-tau protein was found in two athletes, and in one case early-stage CTE. However, following a single moderate-severe TBI neurofibrillary tangles were abundant at ≥1 year although not during the initial post-injury months (Smith et al., 2013) implying that a prolonged injury process is required for tau aggregation. This delayed tau aggregation may be related to axonal injury (Holleran et al., 2017) and persistent neuroinflammation (Cherry et al., 2016, Collins-Praino and Corrigan, 2017).

One limitation of our present study is that the increased tau aggregation has not been validated on neuropathological evaluations. However, in a previous report, an athlete with a history of numerous concussions over 22 years were investigated with the tau PET tracer [^18^F]FDDNP. At autopsy 52 months later, a significant correlation between [^18^F]FDDNP binding and brain tau deposits was observed (Omalu et al., 2018).

We used the [^11^C]PK11195 tracer for evaluation of neuroinflammation/microglial activation (Ramlackhansingh et al., 2011). Although other TSPO PET-ligands such as [^11^C]PBR28 (Murugan et al., 2019) have higher sensitivity than [^11^C]PK11195, the former is confounded by a codominant rs6971 polymorphism and require genotyping of the patients (McKee et al., 2014). In our study where only few rSRC athletes were available for recruitment, a tracer that can be used for all subjects is of importance. The [^11^C]PK11195 tracer binds to TSPO, elevated in activated microglia. The tracer analysis is complicated due to its low extraction and lack of easily identifiable reference tissue. We used supervised clustering and the simplified reference tissue model (SRTM2) with vascular correction (Yaqub et al., 2012), the most widely accepted method for [^11^C]PK11195 data. We observed increased [^11^C]PK11195 binding in e.g. the thalami, internal capsule and medial temporal lobes. These findings are similar to a previous TBI study, where the binding was not correlated with time since injury or extent of structural brain injury (Ramlackhansingh et al., 2011). Of note, TSPO expression and microglia activation may not be mutually dependent following TBI. Using the [^11^C]PBR28 tracer for microglial activation, increased e.g. frontal and temporal white matter binding was observed in TBI, particularly in progressively atrophying white matter tracts (Scott et al., 2018), supporting a link between white matter injury and ongoing inflammation in TBI patients.

In our rSRC cohort, neuroinflammation was less marked than in TBI and increased [^11^C]PK11195 tracer binding was only detected in the medial temporal lobes bilaterally. Future studies are needed to establish any causality between inflammation and cognitive impairment in rSRC. Here, we did not analyze CSF or blood biomarkers of neuroinflammation. In previous studies of mild TBI, levels of several inflammatory cytokines may be persistently elevated (Chaban et al., 2020). However, the dynamics of the neuroinflammatory response to TBI and SRC have not been fully established and any contribution of the inflammatory mediators to the cognitive impairment (Huie et al., 2019) should be addressed in future studies using serial CSF and/or blood sampling.

Of note, the increased [^11^C]PK11195 tracer binding was observed in similar although not identical anatomical regions as the increased [^18^F]THK5317 binding.

In TBI patients, increased flortaucipir binding was observed in both cortical and subcortical distribution, correlating with the degree of white matter damage (Gorgoraptis et al., 2019). In a heterogeneous cohort, a subset of TBI patients had high [^18^F]AV1451 binding associated with reduced white matter tract density (Wooten et al., 2019). In our present study, TBI patients had evidence of widespread white matter injury on MRI. The increased plasma and CSF NF-L levels, a marker of large caliber axon injury, in TBI patients may also indicate a persistent injury process leading white matter atrophy (Johnson et al., 2013, Scott et al., 2018).

While a correlation between closed-head impact and CTE was suggested in numerous reports (Tagge et al., 2018, Kenney et al., 2018), we emphasize that our study is not an investigation of CTE. No investigated rSRC athlete had personality changes, dementia or aggression, traits associated with CTE (Takahata et al., 2019, McKee et al., 2018). To our knowledge, no female athlete with rSRC has been diagnosed with CTE (McKee et al., 2016, Holleran et al., 2017, Stern et al., 2019, Barrio et al., 2015, Dickstein et al., 2016, Bieniek et al., 2015, Shively et al., 2017, Morley, 2018). There were 6 females in our rSRC cohort. In a recent TBI study using [^18^F]AV1451, seven women were investigated of whom 3 had increased tracer uptake (Gorgoraptis et al., 2019). In a study using the [^11^C]PBB3 tracer, only two women with repeated/mild TBI were included (Takahata et al., 2019). Future studies are needed to evaluate potential sex differences in tau aggregation in TBI and rSRC.

Our present study has limitations. We aimed to include patients with prolonged and significant symptoms following rSRCs and recruited athletes from across the country. Thus, only a subset of all SRC athletes met the inclusion criteria and a selection bias cannot be excluded. In view of the heterogeneity of SRC, our results should be interpreted with caution and be confirmed in larger cohorts preferably using SRC athletes from the same sport. Here, only comparison between groups, not at the individual level, can be performed. There may also be differences in the response to TBI and/or rSRC between male and female participants. Furthermore, as in every TBI or rSRC cohort the injury severity may differ within the groups due to the heterogeneity of the injury mechanism. We did also not include inflammatory biomarkers to correlate with the [^11^C]PK11195 PET tracer uptake. Since tau aggregation and/or inflammation may be progressive over time, the variability in time from TBI/last SRC should be considered. While the majority of participants were less than 30 years old, a subset was at the age of 40. While tau aggregations are not normally observed at this age (Johnson et al., 2012), age differences must be considered in tau PET studies. However, the present study is the first to study tau aggregation and neuroinflammation in such young cohorts of two injury severity categories.

In conclusion, we observed increased tau aggregation and neuroinflammation in young TBI patients and in symptomatic rSRC athletes at prolonged post-injury time points. Severe TBI and rSRC and TBI share certain characteristics (McKee et al., 2018); and there were similarities but also important differences in the PET findings of rSRC athletes and TBI patients. These findings imply ongoing pathogenic mechanism that may contribute to the increased risk of neurodegeneration associated with TBI and rSRC.

Previous PET and autopsy studies have evaluated older individuals, with more extensive post-injury times. While many details remain unknown, tau pathology may be the mechanistic link to cognitive symptoms (Takahata et al., 2019) and the tau aggregation observed here may be progressive. Thus, follow-up PET imaging is needed to establish whether tau aggregation and neuroinflammation will progress, be unchanged or diminish over time, and be associated with clinical symptoms.

## Declaration of Competing Interest

The authors declare that they have no known competing financial interests or personal relationships that could have appeared to influence the work reported in this paper.
